# Revealing the role of molecular rigidity on the fragility evolution of glass-forming liquids

**DOI:** 10.1038/ncomms11086

**Published:** 2016-03-30

**Authors:** C. Yildirim, J.-Y. Raty, M. Micoulaut

**Affiliations:** 1 Laboratoire de Physique Théorique de la Matière Condensée, Paris Sorbonne Universités—UPMC, Boite 121, 4, Place Jussieu, 75252 Paris Cedex 05, France; 2 Physique des Solides, Interfaces et Nanostructures & CESAM, B5, Université de Liège, B4000 Sart-Tilman, Belgium

## Abstract

If quenched fast enough, a liquid is able to avoid crystallization and will remain in a metastable supercooled state down to the glass transition, with an important increase in viscosity upon further cooling. There are important differences in the way liquids relax as they approach the glass transition, rapid or slow variation in dynamic quantities under moderate temperature changes, and a simple means to quantify such variations is provided by the concept of fragility. Here, we report molecular dynamics simulations of a typical network-forming glass, Ge–Se, and find that the relaxation behaviour of the supercooled liquid is strongly correlated to the variation of rigidity with temperature and the spatial distribution of the corresponding topological constraints, which ultimately connect to the fragility minima. This permits extending the fragility concept to aspects of topology/rigidity, and to the degree of homogeneity of the atomic-scale interactions for a variety of structural glasses.

## Abstract (web summary)

The fragility describes the degree of slowdown in material dynamics as approaching glass transition temperature, but its molecular origin remains unclear. Here, Yildirim *et al.* show a strong correlation between temperature-dependent spatial distribution of molecular rigidity and the fragility index.

## Graphical abstract

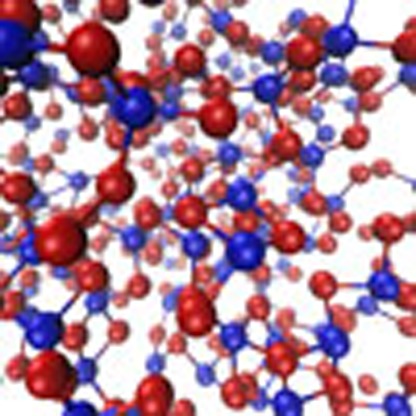

Once the melting point has been bypassed, both viscosity (*η*) and structural relaxation time (*τ*_α_) of a supercooled liquid increase enormously as the temperature is lowered. Fragility (*M*) of glass forming melts has been introduced to quantify such temperature dependences, and a convenient way of obtaining *M*^1^ is to estimate the slope of log_10_(*η*) or log_10_(τ_α_) with temperature, near the glass transition temperature *T*_g_ where, by definition, one has *η*=10^12^ Pa  s, and *τ*_α_∼100 s.


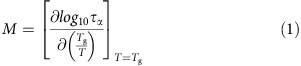


This dimensionless slope *M*, examined for a broad range of glass forming liquids, has been found to vary between a value of 214 (ref. 2) to 14.8 (ref. 3), the latter low value being associated with so-called ‘strong' glass formers, whereas liquids with a high *M* are termed as ‘fragile'. When represented on an appropriate plot (that is, log_10_(*τ*_α_) versus *T*_g_/*T*), strong glass formers (for example, silica) follow an Arrhenius behaviour of the form exp[*E*_A_/*k*_B_*T*], while fragile ones will exhibit an important nonlinear behaviour and a dramatic increase of *η* (or *τ*_α_) over a limited temperature interval once the supercooled liquid approaches *T*_g_. A certain number of correlations between the fragility index *M* and materials properties have been emphasized, and relationships with *T*_g_^2^, structural features^4^,^5^ and elastic properties^6^, have been established. These contributions simply reveal that various aspects of physical properties, structure and interactions influence the glassy relaxation and the fragility.

Here we show by using molecular dynamics (MD) simulations that a typical network-forming system with changing composition displays a certain number of relaxation dynamics anomalies that can be correlated with the strong or fragile nature of the corresponding supercooled liquids. The relaxation properties in network glasses has been quite extensively studied, as in for example, silica^7^. We adopt here, however, a slightly different approach, albeit in line such previous work^7^. Off-stoichiometric glass formers, for example, Ge_*x*_Se_100−*x*_ with GeSe_2_ (*x*=33%) being isochemical to silica, afford, indeed, a unique venue for exploring the dynamics, and understanding ultimately the physical origin of fragility and relaxation in network glasses. First, such studies permit to tune the composition in a continuous fashion, and to compare compositional trends in physical properties, leading to the identification of new correlations. Second, by focusing on a combination of several methods, molecular simulations and rigidity theory^8^,^9^, one can accumulate direct probes and statistical time-dependent quantities that matter during the glass transition. Third, the focus on key physical behaviour, that is, the small temperature evolution of network rigidity for certain compositions is seen to cause the strong character of corresponding glass-forming melts and is driven by the spatially homogeneous distribution of constraints/interactions at select compositions. This offers a new interpretation of the physics driving fragility of network glasses.

It is detected that the compositions displaying an anomalous dynamics merely satisfy the well-known Maxwell stability (isostatic) criterion^10^,^11^, while also weakly depending on temperature. Given that such isostatic compositions also lead to a homogeneous distribution of mechanical constraints at the atomic scale, we argue that fragility deeply connects to the rigidity properties of the underlying network structure, and that fragile glasses are characterized by a spatial distribution of heterogeneous interactions. These conclusions are successfully compared with available experimental data that support the present findings, while also emphasizing the general character of the results for a variety of structural glasses.

## Results

### Dynamic and relaxation anomalies

**Figure 1:**
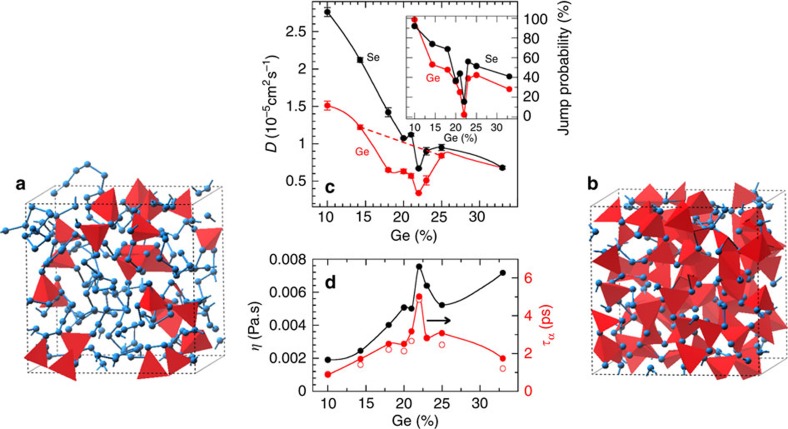
Dynamic and relaxation anomalies in Ge–Se glass-forming melts. At low Ge content, the structure of Ge–Se liquids is made of a Se chains with few GeSe_4/2_ tetrahedral crosslinks (MD snapshots of Ge_10_Se_90_), (**a**) whereas the structure of the stoichiometric GeSe_2_ (**b**) is made of a network of connected tetrahedra. (**c**) Calculated Ge and Se diffusion constants *D* at 1,050 K as a function of Ge content. The inset shows the calculated jump probability that a Ge/Se atom has jumped by a distance *r*>2.88 Å during *t*=20 ps. (**d**) Calculated Stokes–Einstein liquid viscosities *η* and Kohlrausch fitted relaxation time *τ*_α_ of the calculated intermediate scattering function *F*_s_(*k*,*t*) (right axis) at 1,050 K as a function of Ge content. The open symbols (*τ*_α_) refer to the particular value *F*_s_(*k*,*τ*_α_)=1/e. Error bars are in most of the cases of the size of the symbols but some are visible.

To establish our conclusions, we performed First Principles Molecular Dynamics (MD) of Ge_*x*_Se_100−*x*_ liquids and glasses at various Ge content (10≤*x*≤33%). The modelling scheme^12^ reproduces accurately structural data accessed from neutron diffraction in glasses and liquids (Supplementary Figs 1,2 and Supplementary Note 1). The structural changes taking place in Ge–Se are well documented^13^,^14^,^15^, and generic to the family of Group IV chalcogenides, that is, glassy systems made of two-fold Se chains are progressively cross-linked by the addition of four-fold germanium atoms that transforms the basic chain structure into a highly connected network (Fig. 1a,b). The addition of such higher coordinated species leads to a global stiffening of the network structure, and by using the notion of Maxwell rigidity^10^,^11^ and an enumeration of constraints *n*_c_ (refs 8, 9), a flexible-to-rigid transition has been identified at the composition *x*_c_=20%, confirmed experimentally at room temperature^15^, and identified with the mean-field Maxwell isostatic stability criterion *n*_c_=3.

The key findings of our study are shown in Fig. 1c,d. Different quantities encoding the dynamics and relaxation behaviour of the Ge–Se melts (at 1,050 K allowing for a validation of the structural models) have been calculated. From the mean-square displacement *<r*_i_^2^*(t)>*, one obtains the diffusion constants in the long time limit using the Einstein relation *D*=<*r*_i_^2^(*t*)>/6*t*. A global decrease of the Ge and Se diffusivity is obtained with increasing Ge content, in line with the stiffening of the network structure that progressively hinders atomic motion. However, a striking feature is the presence of a narrow interval in composition (typically 18–22% Ge) for which *D*_Ge_ displays an even more marked decrease, about a factor 2–3 less with respect to what would be expected from a smooth decrease of *D*_Ge_ (broken red line). Further characterization of the dynamics and detecting the anomalous behaviour of the Ge diffusivity is provided by the self part of the Van Hove correlation function 4π*r*^2^*G*(*r*,*t*), which represents the probability density of finding an atom at time *t* knowing that this atom was at the origin (*r*=0) at time *t*=0. In fact, a minimum for the corresponding jump probability is found (inset of Fig. 1c) revealing that the atomic motion will be substantially reduced for this particular interval in composition.

### Topological constraints

**Figure 2:**
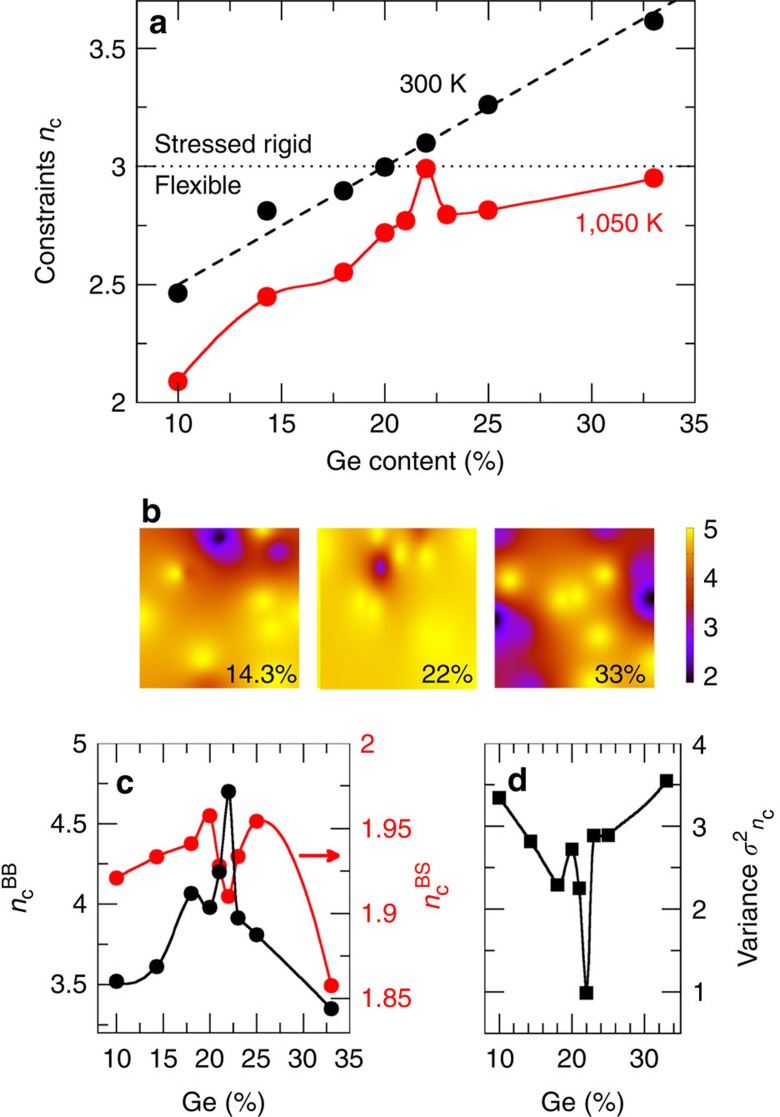
Constraint analysis of Ge–Se melts. (**a**) Global MD constraint count of the liquid (1,050 K), compared with a count in the glassy state (300 K). The broken line represents the Phillips–Thorpe constraint count *n*_c_*=*2*+*5*x* (ref. 9). (**b**) Contourplots of the Ge BB constraint distribution among the structure for selected compositions. They have been generated by focusing on the constraint distribution inside a slab of 3.2±0.2 Å. (**c**) Calculated Ge BB (black) and BS (red, right axis) constraint density as a function of Ge content. (**d**) Variance *σ*_*n*c_ of the Ge BB constraint population.

A central result is the detection of the crucial role played by rigidity in the anomalous relaxation dynamics of Fig. 1, and the indication of a fragility minimum. Within temperature-dependent rigidity theory^18^, the assumption of an Adam–Gibbs form for the relaxation time *lnτ_α_*∝*1/TS*_c_, and the contribution of the number of topological degrees of freedom *f(x,T)=3−n*_c_*(x,T)* to the configurational entropy^17^ *S*_c_*(x,T)* leads to the prediction of the fragility index *M*:


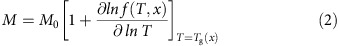


which depends only on the scaling of *f(x,T)* with temperature. The validity and predictive power of equation (2) has been checked on a certain number of glasses by building structural models, establishing their constraint count, and comparing with a composition dependence of fragility measurements such as for borates^18^ or phosphates^19^. MD-based constraint counting algorithms (Supplementary Note 2) are used^20^ to yield *n*_c_*(x,T)* from Ge/Se bond-stretching (BS) and bond-bending (BB) motions, and they show that (i) for the 300 K glasses, *n*_c_ follows exactly the mean-field estimate *n*_c_*=*2+5*x* (Fig. 2a)^8^,^9^, leading to an isostatic condition (*n*_c_=3) for ∼20% Ge that agrees with the experimentally measured threshold value^15^, and, importantly, (ii) the calculated *n*_c_*(x,T)* displays a minimum change with temperature for 20–22% Ge. The obtained minimum change is robust (see Supplementary Note 3). Following equation (2), this leads to a minimum for the liquid fragility with composition given the weaker variation of the term *dn*_c_*/d*ln*T* at *x*∼22%, and coincides with *n*_c_=3.

### Viscocity anomaly

**Figure 3:**
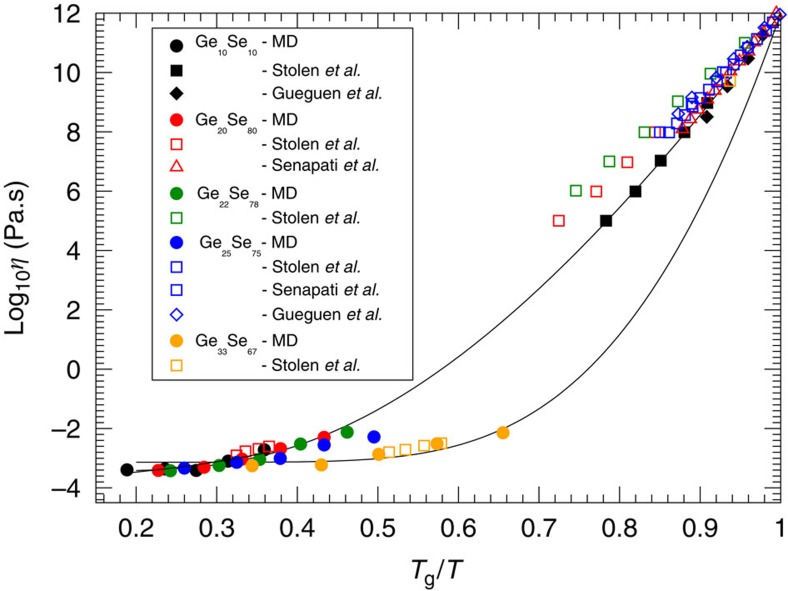
Comparison between estimated and measured viscosities. Comparison between the Stokes–Einstein calculated (filled symbols) viscosities at various Ge–Se compositions and experimental measurements of viscosity (open symbols) from Stølen *et al.*^22^ Senapati *et al.*^23^ and Guegen *et al.*^24^ Note that only Stølen *et al.* have investigated the high temperature region where numerical data can be directly compared. The solid lines correspond to fits using the Mauro–Yue–Ellison–Gupta–Allen (MYEGA) equation^25^ that describes with an increased accuracy the high temperature viscosities as compared with alternative fitting functionals^26^ (for example, Vogel–Fulcher–Tamman, VFT).

Independent support for this conclusion is given by a Stokes–Einstein estimate of the liquid viscosity. Given the system size (250 atoms), and First Principles MD timescale (ps), a direct calculation of the Green–Kubo viscosities is out of reach. However, the validity of the Stokes–Einstein *η*=*k*_B_*T*/6π*rD* where 2*r*=*ρ*^−1/3^ involves the liquid densities *ρ*, can been established from calculated diffusivities for the supercooled state, similarly to a previous application^21^, and this provides a validation of the dynamic behaviour of the models (see Supplementary Note 4). Next, one can derive from the calculated diffusivities *D*_Ge_*(x,T)* (Fig. 1a) corresponding viscosities using the Stokes–Einstein relationship. Results using such calculated viscosities are shown in Fig. 3, and a direct comparison in an Angell plot represents calculated and measured log_10_(*η*) as a function of *T*_g_/*T*. Note that we have checked that the Stokes–Einstein remains valid for the considered range of temperatures. The agreement is found to be very good for the two compositions for which a direct comparison is possible (Ge_20_Se_80_ and Ge_33_Se_67_). Having validated the approach, we then use it to extract the viscosity *η*(*x*,1,050 K) along the 1,050 K isotherm (Fig. 1d). A maximum for *η* is obtained that correlates close to the isostatic composition (Fig. 1d), with the maximum of the structural relaxation time *τ*_α_ calculated from the α-relaxation regime of the intermediate scattering function (Supplementary Note 5 and Supplementary Fig. 5). This provides an additional indication that strong glass-forming liquids with a minimum fragility are obtained for the 22% composition.

## Discussion

When the details of the constraint contributions are investigated, and their spatial distribution analysed (Fig. 2b), additional important features can be linked to the weak variation of *n*_c_ with temperature. First, for the composition at which the weakest change in *n*_c_ is found (22%), the BB constraints are nearly homogeneously distributed in the structure given that *n*_c_^BB^ maximizes to its nearly low temperature value (*n*_c_^BB^=5), which reduce the possible fluctuations (Fig. 2c). This contrasts significantly with all the other compositions (for example, 14.3 and 33%, Fig. 2b), which display a heterogeneous distribution of constraints broken by thermal activation (Fig. 2d). The variance of *n*_c_^BB^ is also a measure of the spread in the spatial distribution of atomic interactions leading to rigid constraints. For the compositions in the region 20–23%, one now acknowledges that homogeneity of stress (that is, interactions) will not only induce a cascade of dynamic anomalies as detected from the diffusivity minima and viscosity/relaxation time maxima located in the same range of compositions (Fig. 1) but also represents a more stable state at the nanoscale. As a consequence, thermal changes must lead to minor changes of *n*_c_ with temperature, and ultimately will minimize fragility (equation (2)). In this particular region of anomalous behaviour, one furthermore detects weak effects on structure with chemical composition given that both the pair correlation function *g*(*r*) in real space and the structure factor in reciprocal space exhibit small differences with Ge content (see Supplementary Note 1 and Supplementary Figs 1,2). For instance, the structural properties of the 22% composition that appears to be so particular in terms of dynamic properties (Fig. 1), are found to be nearly identical to the 20% one, although their relaxation time differs by a factor 2 (Fig. 1b) and their rigidity also varies substantially within a tiny compositional interval. This, ultimately, emphasizes the predominance of rigidity and its spatial distribution on the liquid relaxation properties, over aspects of structure.

**Figure 4:**
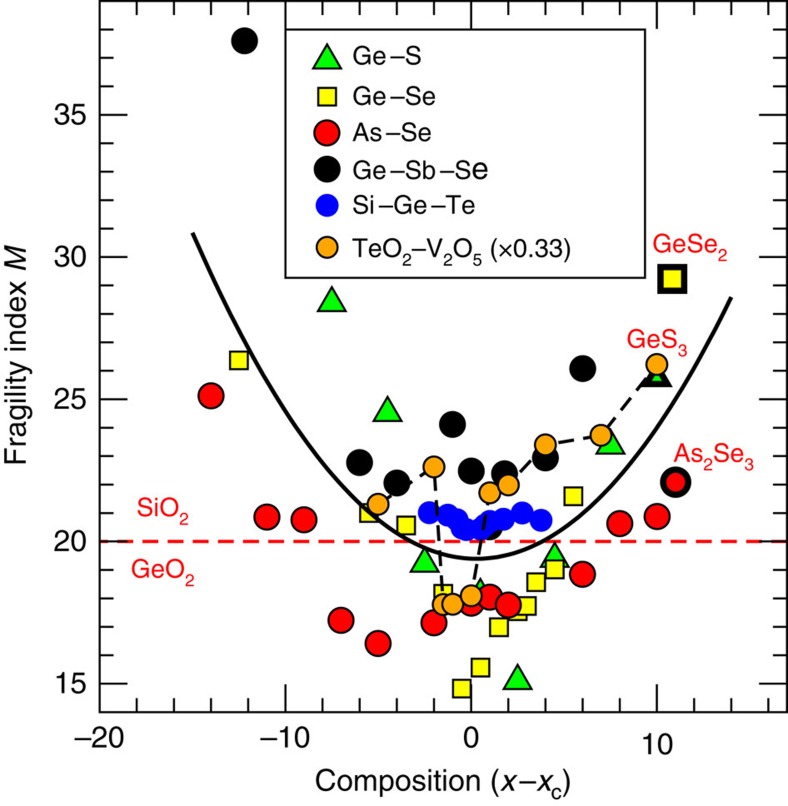
A summary of experimentally measured fragilities in glass network-forming melts. Experimental fragilities as a function of a rescaled composition corresponding to the centroid *x*_c_ of the corresponding isostatic window (or Boolchand phase): Ge–S^27^, Ge–Se^3^, As–Se^28^, Ge–Sb–Se^29^, Si–Ge–Te. All display a minimum at or close to *x=x*_c_. Note that the minimum for Ge–Si–Te is clearly visible if represented on an appropriate scale^30^. The trend in composition for TeO_2_–V_2_O_5_ is highlighted by a thin broken line (P. Boolchand, unpublished results). The solid line is a global quadratic fit to the whole data sets, and serves only as guide. Stoichiometric compounds are signalled.

This physical picture revealing the central role played by network rigidity is consistent with conclusions drawn from a compilation of fragility data on network glasses including chalcogenides and oxides for which the location of the isostatic composition is determined from calorimetric measurements^3^,^27^,^28^,^29^,^30^. These liquid fragilities are represented in Fig. 4, and show that once the composition is rescaled with respect to the centroid *x*_c_ of the isostatic composition interval (for example, *x*_c_=22.5% in Ge_*x*_Se_100−*x*_; ref. 3), all the data exhibit a minimum in *M* at *x∼x*_*c*_. This not only emphasizes that rigidity affects the fragility evolution of melts, a qualitative correlation that has been reported in the literature for quite some time (ref. 3) and reference therein), but from Fig. 2b, one also realizes that the spatial distribution of atomic scale interactions/constraints is a key feature for the understanding of transport properties during the glass transition. This conclusion can be only drawn from a detailed inspection of constraints, accessed from MD on individual atoms. Although further detailed analysis on the constraint behaviour of, for example, organic glass formers is necessary, these results may indicate that liquids with non-directional bonding and, therefore, with a more probable heterogeneous distribution of interactions will lead to a much more fragile behaviour.

## Methods

### First-principles molecular dynamics simulations

Ge–Se liquids and glasses have been investigated using Car–Parrinello molecular dynamics simulations. The system contained 250 atoms, and up to nine compositions Ge_*x*_Se_100−*x*_ have been simulated in NVT ensemble with cubic cells of sizes allowing to recover the experimental density^31^,^32^ of corresponding liquids and glasses (that is, 19.97 Å for GeSe_9_).

Density functional theory has been used to describe the electronic structure that evolved self-consistently with time. We have adopted a generalized gradient approach using the exchange energy obtained by Becke^33^ and the correlation energy according to Lee, Yang and Parr (LYP)^34^. The BLYP approach was used due to its account on valence electron localization effects, and an increased agreement with experiments on structure as revealed by a previous study^12^,^35^. Valence electrons have been treated explicitly, in conjunction with norm conserving pseudopotentials of the Trouiller–Martins type to account for core–valence interactions. The wave functions have been expanded at the Γ point of the supercell on a plane-wave basis set having an energy cutoff *E*_c_=20 Ry which is a standard value for the investigation of chalcogenides^36^. A fictitious electron mass of 200 a.u. was used in the first-principles molecular dynamics approach. The time step for integrating the equations of motion was set to Δ*t*=0.10 fs. The temperature control was achieved for both ionic and electronic degrees of freedom using Nosé–Hoover thermostats. The initial coordinates of 250 atoms have been constructed using the atomic positions in a GeSe crystal. To achieve the correct compositions, Ge atoms were then replaced with Se atoms in an appropriate fashion, depending on the target composition.

We carried out simulations at *T*=2,000 K for a period of 22 ps to lose the memory of initial configuration, and then investigated a certain number of isotherms (for 25–30 ps each): 2,000 K, 1,600 K, 1,373 K, 1,200 K, 1,050 K, 800 K and 300 K. The first 2 ps at all the temperatures have been discarded. The 300 K trajectories result from three independent quenches with starting temperature 1,050 K.

Certain compositions have been particularly checked, for example, the 22 and 23%, and a series of five trajectories at 1,050 K obtained from five independent high-temperature liquid starting configurations at 2,000 K have been analysed. The statistical average calculated quantities (for example, *n*_c_, *n*_c_^BB^, *D*_Se_, and so on) have been found to be consistent (see Supplementary Note 3). Similarly, effects of size (480 atoms and the present 250 atoms) and excursion in density have been tested and have confirmed that the constraint count does not depend on these different simulation conditions. For instance, for the 23% composition, it has been found that *n*_c_=2.84 for *N*=480, a value that is very close to the value found for *N*=250 (*n*_c_=2.79).

## Additional information

**How to cite this article:** Yildirim, C. *et al.* Revealing the role of molecular rigidity on the fragility evolution of glass-forming liquids. *Nat. Commun.* 7:11086 doi: 10.1038/ncomms11086 (2016).

## Supplementary Material

Supplementary Information Supplementary Notes 1-5 and Supplementary References
