# Supplementary material for: Revealing the role of molecular rigidity on the fragility evolution of glass-forming liquids
Source: Nat Commun. 2016 Mar 30;7:11086. doi: 10.1038/ncomms11086 (PMC4820934; doi:10.1038/ncomms11086)
Supplement: Supplementary Information — Supplementary Notes 1-5 and Supplementary References [file ncomms11086-s1.pdf]

Supplementary figures

***"Revealing the crucial role of molecular rigidity on the fragility evolution of glass-forming melts"*, by C. Yildirim, J.-Y. Raty and M. Micoulaut**

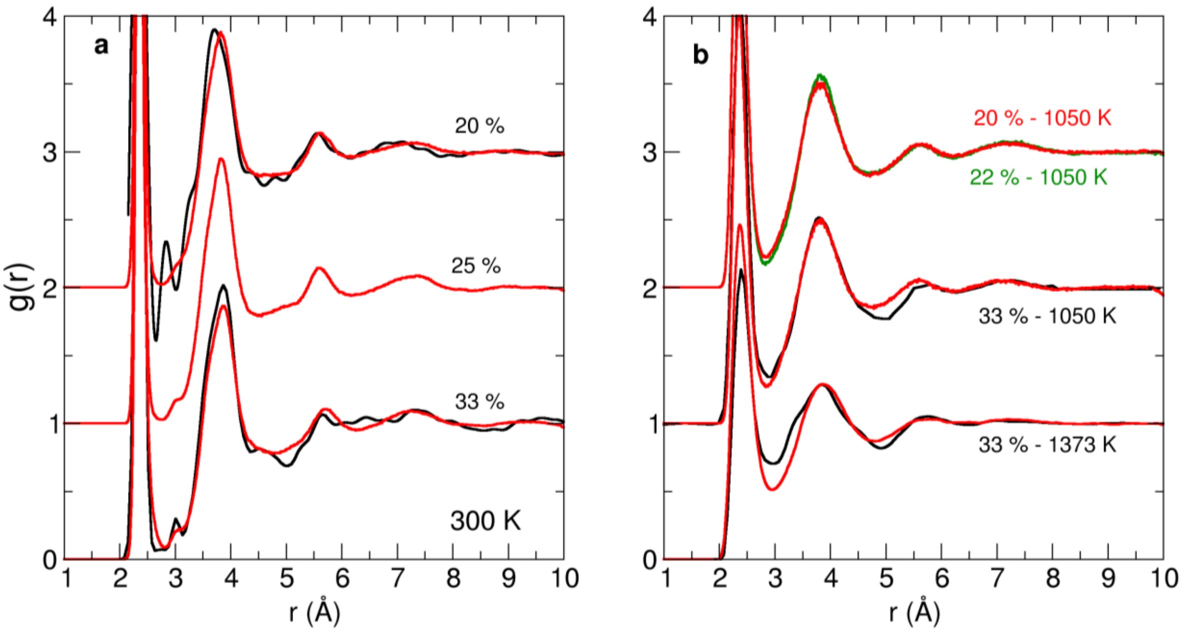

Supplementary Figure 1: Comparison between calculated and measured pair correlation functions. Pair correlation function *g(r)* in Ge-Se glasses (a) and melts (b) for different Ge content (red), and compared to available measurements from neutron diffraction (black)[1] at 300 K, 1050 K and 1373 K. The green curve corresponds to the pair correlation function of $Ge_{22}Se_{78}$.

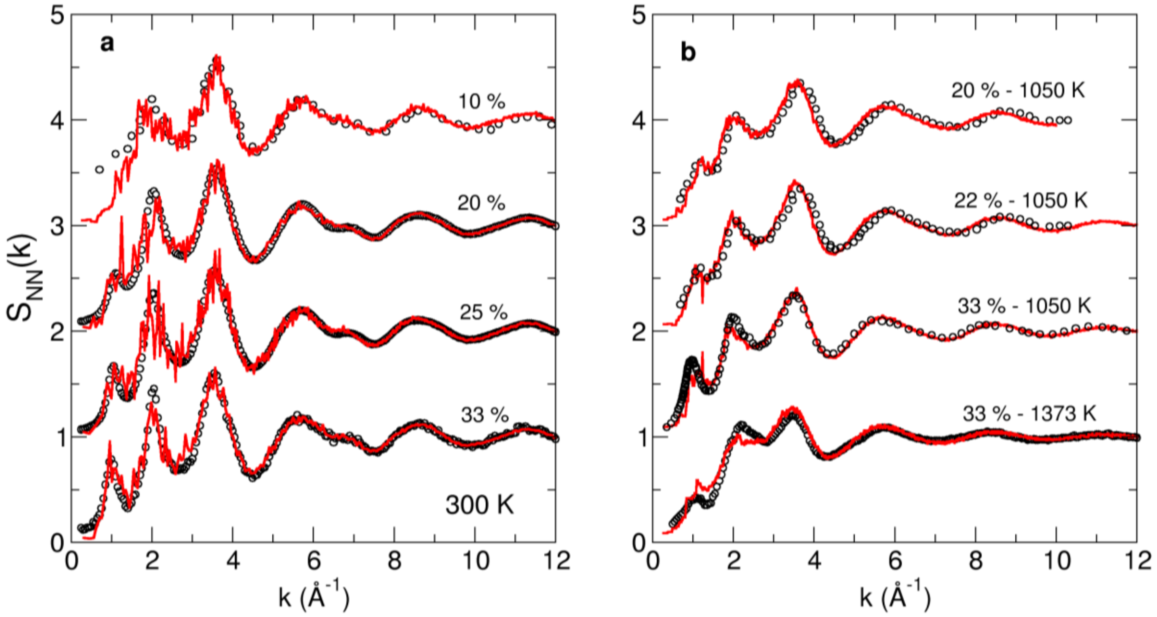

Supplementary Figure 2: Comparison between calculated and measured structure factors. Bhatia-Thornton structure factor $S_{NN}(k)$ in Ge-Se glasses (a) and melts (b) for different Ge content (red), and compared to available measurements (circles) from neutron diffraction: $Ge_{10}Se_{90}$ [2], $Ge_{20}Se_{80}$ [3], all other compositions/temperatures [1]. The calculated structure factor of $Ge_{22}Se_{78}$ is compared to experimental results of $Ge_{20}Se_{80}$ [3].

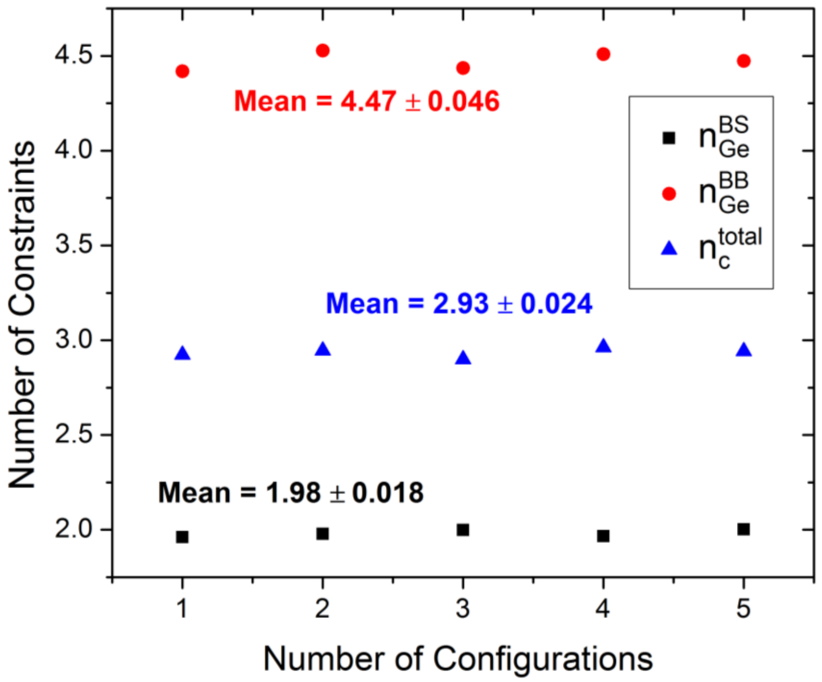

Supplementary Figure 3: Statistical analysis of constraint calculation. Number of constraints n_c^{Total} and Ge related constraints calculated for five independent configurations at 1050 K for the 22% system.

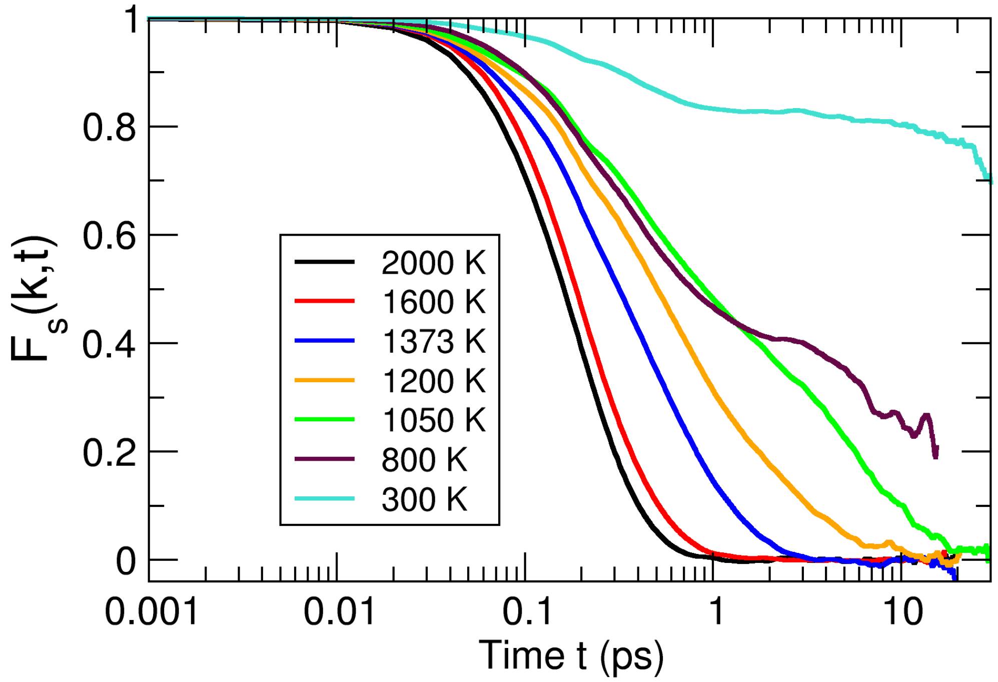

Supplementary Figure 4: Temperature effect on the intermediate scattering function. Time dependence of $F_s(k,t)$ of $Ge_{20}Se_{80}$ at different temperatures showing the single step relaxation process at high temperature (2000 K), and the onset of a two-step relaxation process for T<1050 K. For the present purpose, the isotherm 1050 K is the lowest temperature that leads to a diffusive regime, and that can be used for fitting the α-relaxation to obtain the relaxation time τ_α (Fig. 1b).

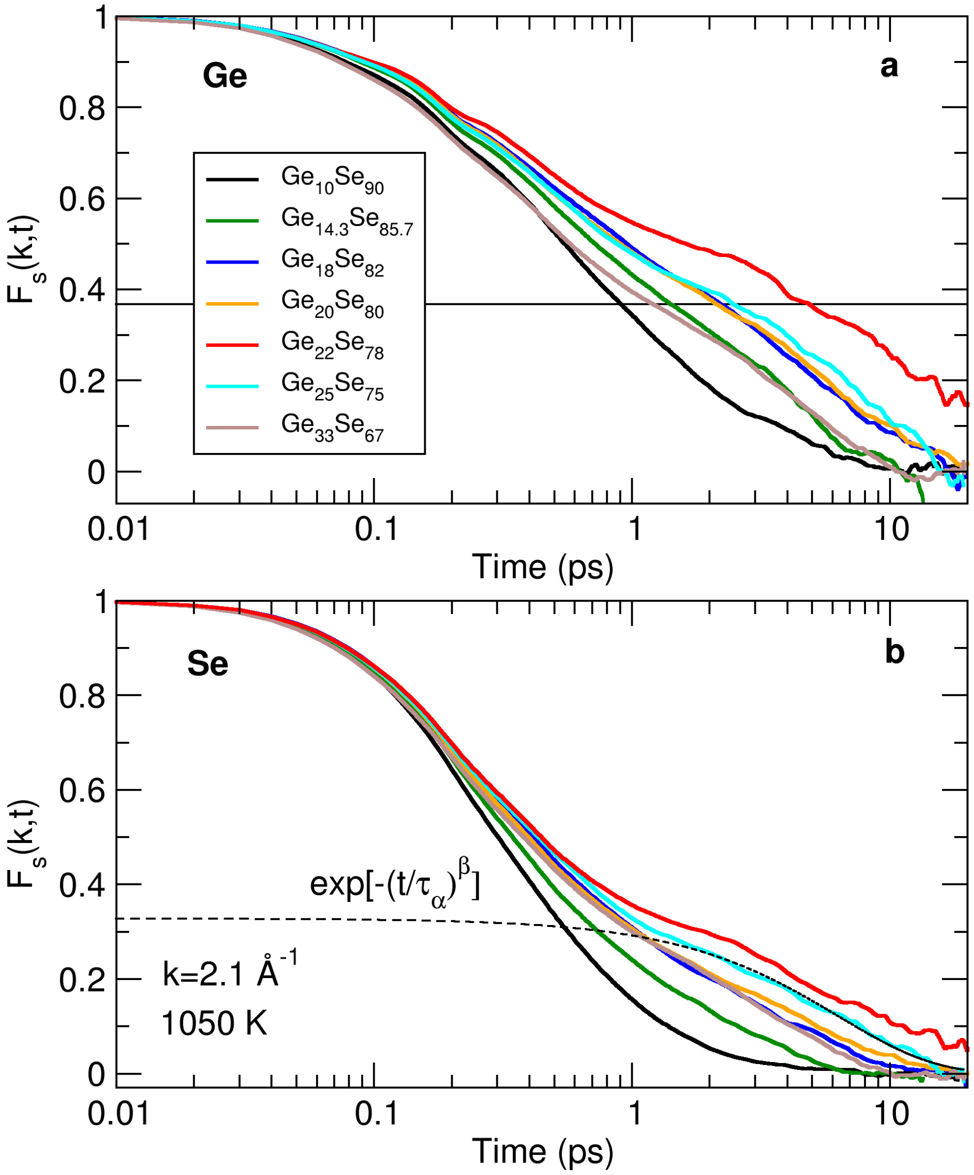

Supplementary Figure 5: Effect of composition on the intermediate scattering function. Time dependence of $F_s(k,t)$ for select different compositions. The thin solid line represents *1/e*, and Kohlrausch exponential fits (broken line) have been performed in the long time limit, i.e. when $F_s(k,t)<1/e$.

Supplementary notes

Supplementary note 1 Validation of the structural models.

Prior to the investigation of the dynamics, we have checked that our structural models were realistic enough. In the glass (300 K), a certain number of quantities can be directly compared to results from neutron diffraction[1], including the pair correlation functions *g(r)* (Supplementary Figure 1) and structure factors *S(k)* (Supplementary Figure 2).

In real space, the computed total pair distribution function g(r) for the Ge_xSe_{1-x} glasses and liquids compare very convincingly to the experimental counterparts (Supplementary Figure 1)[1,2]. All the features of the pair distribution function are reproduced such as the main peak at 2.36 Å, (experimentally[1], one has 2.35 Å) and the secondary peak at 3.85 Å which is due to the Se-Se distance defining the edge of the $GeSe_{4/2}$ tetrahedra. The large intensity of the first peak, which mostly arises from the Ge-Se bond distance, actually overwhelms in Ge-rich glasses other contributions due to Ge-Ge and Se-Se correlations in the structure, and which has been investigated from a detailed analysis of the partial pair correlation functions[1].

Regarding the reciprocal space, an excellent overall agreement of the calculated $S_{NN}(k)$ with the experimental counterpart is found (Supplementary Figure 2), except for a notable underestimation of the main peaks amplitude at the highest temperatures. All typical peaks of the diffraction pattern are reproduced over the entire range of wave vectors *k*: the first sharp diffraction peak (FSDP) at k =1.0 Å $^{-1}$, the first principal peak at 2.1 Å $^{-1}$, and the second principal peak at 3.6 Å $^{-1}$ The higher wavevector region (k= 10–12 Å $^{-1}$) is also very well reproduced, including a small shoulder peak at around 6.5 Å $^{-1}$. This agreement is verified for several available compositions in the glassy state (300 K) and for certain liquid temperatures (1050 K and 1373 K). It is to be noticed that small changes in composition (e.g. 1% Ge) do not change substantially the calculated diffraction pattern. For instance, the functions *g(r)* and $S_{NN}(k)$ of $Ge_{21}Se_{79}$ and $Ge_{22}Se_{78}$ (green curve in Supplementary Figures 1 and 2) are found to be very close to the one of $Ge_{20}Se_{80}$. Therefore, the difference in dynamic properties between 20 and 22% Ge (Fig. 1) cannot rely on structural changes.

Supplementary note 2 MD based constraint counting

In order to evaluate the number of topological constraints represented in Fig. 2, we have calculated the radial and angular excursions between pairs or triplet of atoms, based from the MD atomic configurations at fixed Ge content and temperature. This enumeration is directly inspired by the classical mechanics view of mechanical constraints associating large/small radial or angular motion with the absence/presence of corresponding bond-stretching (BS) and bond-bending (BB) restoring forces. It has been shown[4,5] that BS constraints can be simply enumerated from the coordination number r_i of the atoms, and according to Phillips and Thorpe, this leads to a contributions of $r_i/2$ for the BS constraint, each bond/interaction being shared by two neighbors. To derive angular (BB) constraints, one follows the angular motion around each individual atom k (k=Ge,Se) defined by a set of two neighbors. Over the time MD trajectory, the corresponding bond angle distribution $P_k(\theta)$ allows defining a mean (the first moment of $P_k(\theta)$) and a standard deviation σ_k (the second moment) that shows a bimodal distribution $f(\sigma_k)$ for the various considered conditions. Atoms subject to a rigid bending interaction contribute to the part of $f(\sigma_k)$ with low σ_k ($\sigma_k<\sigma_{min}$) and have a corresponding angle that acts as a rigid BB constraint, inducing network stiffening. The threshold value σ_{min} is fixed by $f(\sigma_{min})$ minimum. Averages over the whole simulation box then lead to the mean number of constraints n_c^{BB} per atom that is shown in e.g. Fig. 2c.

Supplementary note 3 Cross-checking certain important compositions

For the compositions in the region 20-25%, we have realized a certain number of additional tests of validation in order to check that the results do not depend on e.g. system size or initial configuration. Five independent configurations have been chosen in the high temperature liquid and cooled to the isotherm of 1050 K over the same time interval (20 ps). All led to similar results in terms of constraints, either total number of interaction or related numbers (BS or BB) with a minimal spread between configurations. Supplementary figure 3 shows the calculated values for such configurations which validate e.g. the data point of 22%

represented in Fig. 2a, c and d. In addition, the calculated pair correlation function for the five independent configurations at 1050 K of the 22% system lead to identical results (Supplementary figures 1 and 2), and corresponding *g(r)* are very close to the 20% system.

Supplementary note 4 Validation of the dynamic behavior

In order to focus on the transport properties, one computes first the mean-square displacement of a tagged atom of type α in the Ge-Se liquids:

$$\langle r^2(t)\rangle = \frac{1}{N_\alpha}\sum_{i=1}^{N_\alpha}\langle|\boldsymbol{r}_i(t) - \boldsymbol{r}_i(0)|^2\rangle$$

(Supplementary equation 1)

with N_α is the number of atoms α. In a log-log plot of the mean square displacement with time, the diffusive regime can be detected from a linear behaviour with a slope of one in the long time limit, and such a regime is detected for the 1050 K liquid in $GeSe_2$ for t > 5ps. The diffusion constant D can be obtained via the Einstein relation limit from $D=<r^2(t)>/6t$.

We are not aware of any diffusivity data for Ge-Se melts. However, in order to check for the accuracy of our models, we can estimate an approximate diffusion constant from viscosity data using the Stokes-Einstein relation $\eta=k_BT/6\pi rD$ where $2r=\rho^{-1/3}$ involves the liquid densities ρ [6]. Note that a similar relationship (Eyring), $D=k_BT/\lambda\eta$, where λ is a typical hopping length for the diffusing atom can be used[7]. In silicate liquids e.g., Na_2SiO_3, this Eyring equation holds very well with λ=2.8 Å, a distance typical of Si-Si and O-O separation in these melts, and this conclusion has also been demonstrated from a separate estimate of diffusivities and Green-Kubo viscosities using molecular simulations[8].

From experimental viscosity data of Ge-Se melts, one is able to proceed in a similar way and deduce such approximate diffusivities from the Stokes-Einstein equation, i.e. we obtain a direct comparison between our simulated diffusivities and experimental viscosity determined diffusivities. For instance, for the temperature 1373 K, a diffusion constant equal to 4.70×10^{-5} $cm^2.s^{-1}$ can be calculated for $GeSe_2$, which compares favorably (same order of

magnitude) to the calculated one from viscosity data (~0.002 Pa.s) of Stolen (~1300 K[9]) which leads to D_{exp}=3.2x10^{-5} cm^2.s^{-1}.

Supplementary note 5 Relaxation behavior

The relaxation behaviour of the Ge-Se liquids has been investigated by analyzing the time dependence of the intermediate scattering function $F_s(k,t)$ at a wavevector (2.10 Å^{-1}) corresponding to the main peak of the static structure factor $S(k)$ (Supplementary Figure 2). This function follows the Fourier components of density correlations and can be investigated for different conditions (Supplementary Figures 4 and 5). For a fixed composition and at low temperature (T<800 K), $F_s(k,t)$ exhibits the usual relaxation behavior with a β-relaxation plateau (e.g. at $F_s(k,t)$~0.2 for T=800 K in $Ge_{20}Se_{80}$, Supplementary Figure 4), followed by an α-relaxation behavior, detectable only for T>800K. This trend is similar to many other glass-forming systems including silica[10].

The same relaxation behavior can be investigated as a function of composition along an isotherm (1050 K, Supplementary Figure 5), and it is seen that composition does not affect the time evolution of the different intermediate scattering functions in a monotonic fashion since e.g. $Ge_{22}Se_{78}$ displays the slowest time evolution for $F_s(k,t)$ exhibiting even a starting β-relaxation plateau (e.g. at $F_s(k,t)$~0.5 in Supplementary Figure 5), followed by an α-relaxation behavior, also detectable at long times for all compositions. In the α-relaxation regime, (typically when $F_s(k,t)<e^{-1}$), the behavior of F_s(k,t) can be fitted with a Kohlrausch stretched exponential function ($exp[-t/\tau_\alpha)^\beta]$, broken line) to yield the structural relaxation time τ_α with composition (Fig. 1d, right axis).

Supplementary references

1. P.S. Salmon, *Structure of liquids and glasses in the Ge-Se binary system*. *J. Non-Cryst. Solids* **353**, 2959-2974 (2007).
2. N. Ramesh Rao, P. S. R. Krishna, S. Basu, B. A. Dasannacharya, K. S. Sangunni, E. S. R. Gopal*, Structural correlations in* Ge_xSe_{1-x} *glasses – a neutron diffraction study,* J. Non-Cryst. Solids **240**, 221 (1998).
3. M.J. Haye, C. Massobrio, A. Pasquarello, A. De Vita, S.W. De Leeuw, R. Car, *Structure of liquid* Ge_xSe_{1-x} *at the stiffness transition*, Phys. Rev. B **58**, R14661 (1998).
4. M. Bauchy, M. Micoulaut, M. Celino, M. Boero, S. Le Roux, C. Massobrio, *Angular rigidity in tetrahedral network glasses with changing composition*, Phys. Rev. B **83**, 054201 (2011).
5. M. Bauchy, M. Micoulaut, *Transport anomalies and adaptative pressure-dependent topological constraints in tetrahedral liquids : evidence for a reversibility window analogue,* Phys. Rev. Lett. **110**, 095501 (2013)
6. J. Y. Raty et al., Distance correlations and dynamics of liquid GeSe: An ab initio molecular dynamics study, Phys. Rev. B **64**, 235209 (2001).
7. B.O. Mysen, P. Richet, *Silicate melts and glasses*, Elsevier, 2005
8. M. Bauchy, B. Guillot, M. Micoulaut, N. Sator, *Viscosity and viscosity anomalies in model silicates and magmas: a numerical investigation*, Chem. Geol. **346**, 47 (2013).
9. S. Stølen, T. Grande, H.-B. Johnsen, Fragility transition in $GeSe_2$–Se liquids, Phys. Chem. Chem. Phys. **4**, 3396 (2002)
10. J. Horbach, W. Kob, *The relaxation dynamics of a viscous silica melt: II The intermediate scattering functions*, Phys. Rev. E **64**, 041503 (2001)
